# SARS-CoV-2 infection in households with and without young children: Nationwide cohort study, Denmark, 27 February 2020 to 26 February 2021

**DOI:** 10.2807/1560-7917.ES.2022.27.32.2101096

**Published:** 2022-08-11

**Authors:** Anders Husby, Giulia Corn, Tyra Grove Krause

**Affiliations:** 1 Department of Epidemiology and Biostatistics, Imperial College London, London, United Kingdom; 2 Department of Epidemiology Research, Statens Serum Institut, Copenhagen, Denmark; 3 Department of Infectious Disease Epidemiology and Prevention, Statens Serum Institut, Copenhagen, Denmark

## Abstract

**Background:**

Infections with seasonally spreading coronaviruses are common among young children during winter months in the northern hemisphere; the immunological response lasts around a year. However, it is not clear if living with young children changes the risk of SARS-CoV-2 infection among adults.

**Aim:**

Our aim was to investigate the association between living in a household with younger children and the risk of SARS-CoV-2 infections and hospitalisation.

**Methods:**

In a nationwide cohort study, we followed all adults in Denmark aged 18 to 60 years from 27 February 2020 to 26 February 2021. Hazard ratios of SARS-CoV-2 infection by number of 10 months to 5 year-old children in the household were estimated using Cox regression adjusted for adult age, sex and other potential confounders. In a sensitivity analysis, we investigated the effect of the children's age.

**Results:**

Among 450,007 adults living in households with young children, 19,555 were tested positive for SARS-CoV-2, while among 2,628,500 adults without young children in their household, 110,069 were tested positive for SARS-CoV-2 (adjusted hazard ratio (aHR) = 1.10; 95% confidence interval (CI): 1.08–1.12). Among adults with young children, 620 were hospitalised with SARS-CoV-2, while 4,002 adults without children were hospitalised with SARS-CoV-2 (aHR = 0.97; 95% CI: 0.88–1.08). Sensitivity analyses found that an increasing number of younger children substantially increased the risk of SARS-CoV-2 infection but not hospitalisation.

**Conclusion:**

Living in a household with young children was associated with a small increased risk of SARS-CoV-2 infection.

## Introduction

Severe acute respiratory syndrome coronavirus 2 (SARS-CoV-2) is emerging as a common respiratory infection worldwide, with infections resulting from a combination of viral antigenic drift and waning immunity from previous infections and vaccinations [1]. However, although the relationship between the age of household members and the risk of SARS-CoV-2 infection has been studied intensely in contact tracing studies [2,3], the association is not clear on the population level [4]. The related seasonally spreading human coronaviruses (HCoV), e.g. OC43 and NL63, are particularly prevalent among young children during the winter months [5], and SARS-CoV-2 infections might likewise result specifically from household exposure to younger children. On the other hand, previous human coronaviruses (HCoV) infections among close contacts of younger children might result in some protection from SARS-CoV-2 infection or reduce SARS-CoV-2 severity [6].

To explore the association between living in households with younger children and the risk of SARS-CoV-2 infection and SARS-CoV-2 hospitalisation, we took advantage of complete individual-level data on inhabitants of all households in Denmark and nationwide information on all laboratory-confirmed SARS-CoV-2 infections and hospitalisations. Using this information, we quantified the role of age and number of household children on adult SARS-CoV-2 infection risk.

## Methods

### Materials

The Danish Civil Registration System provides demographic information on the Danish population, in addition to information on household members and number of children [7]. Information on PCR tests for SARS-CoV-2 is available through MiBA, the Danish Microbiology Database, which includes all microbiological test results from public laboratories in Denmark [8]. The study period was from 27 February 2020 to 26 February 2021 and covered predominantly a time window without accessible vaccines and with circulation of the ancestral SARS-CoV-2 variant. Denmark had one of the highest SARS-CoV-2 PCR testing capacities in Europe during the study period, with free PCR-tests performed by medical professionals easily accessible and offered to all inhabitants, regardless of symptoms, through the public healthcare system [9]. Information on all hospitalisations in Denmark is available through the Danish National Patient Registry [10]. 

### Study population

All adults aged 18 to 60 years living in Denmark on 1 January 2020 with known address were included in the study cohort. In addition, we constructed a cohort of all SARS-CoV-2 PCR test-positive individuals, aged 18 to 60 years, who were followed up for hospitalisation until 30 days after positive test.

We excluded adults living in households with seven or more individuals (only 1.7% of the population in Denmark), to avoid inclusion of households consisting of multiple families (e.g. collective housing communities living at the same address).

### Exposure

The primary exposure was defined as living, per 1 January 2020, in a household with one or more children aged 10 months to 5 years. In this age span, children are usually enrolled in childcare institutions [11] and seroconvert against seasonal coronaviruses [12-14]. The exposure is therefore a proxy of recent close contact with a child infected with HCoV, as used previously [6]. To ensure validity of a close relationship between adult and children, only individuals who were legal parents to the child or children were included as exposed in the primary exposure analysis. In the sensitivity analysis we examined other types of co-residence.

### Study covariates

In addition to age and sex, we adjusted for urbanicity by grouping the 98 Danish municipalities into 10 groups based on population density, from most rural to most urban. We further adjusted for ethnicity (based, in accordance with definitions used by Statistics Denmark, on information on the country of birth of the cohort members and the country of birth of their parents), as an earlier report found higher incidence of SARS-CoV-2 infection among migrant groups in Denmark [15]. Rare cases of missing ethnicity were defined as Danish ethnicity. Furthermore, we adjusted for the following comorbidities: asthma, chronic pulmonary disease (incl. chronic obstructive pulmonary disease), cardiovascular disease, diabetes mellitus, inflammatory bowel disease, malignancy and renal failure (see definition of comorbidities in Supplementary Table S1). All covariates were determined as per 1 January 2020.

### Outcomes

The main outcome was the first positive SARS-CoV-2 PCR test from 27 February 2020 (the date of the first positive SARS-CoV-2 test in Denmark) to 26 February 2021. As secondary outcome we investigated risk of hospitalisation within 30 days after the first positive SARS-CoV-2 PCR test. See Supplementary Figure S1 for an overview of daily SARS-CoV-2 test positivity rate, cases and hospital admissions during the study period.

### Statistical analysis

Hazard ratios for SARS-CoV-2 infection by household type were estimated using Cox regression with calendar period as the underlying time scale, adjusting for sex and adult age. In all analyses, a robust variance structure that clustered observations by household membership was used to adjust standard errors. Thereby, the analyses took into account correlation between adults living in the same household. Cohort members were followed from 27 February 2020 until outcome of interest, death, emigration or until 26 February 2021, whichever came first. In a secondary analysis, we investigated the 30-day hazard ratio of hospitalisation among individuals testing positive for SARS-CoV-2 by household type.

In sensitivity analyses, we investigated interaction with adult age, sex, and period of testing (27 February to 26 March (before lockdown), 27 March to 28 April (first lockdown), 29 April to 30 June (early reopening), 1 July to 30 November (late reopening), 1 December 2020 to 26 February 2021 (second pandemic wave)). Furthermore, we investigated the effect of different age criteria for the exposure definition, different types of households based on co-residence and legal parenthood, and different types of households with and without co-residence of young and older children. In addition, we estimated the effect of number of adults in the household.

To investigate whether adults living with young children were tested more often than other adults, we compared the incidence rate ratio of SARS-CoV-2 PCR testing within the latest 60 days among the two groups using Poisson regression. All tests until the first positive test, if any, were included in the model as outcome.

Finally, using an alternative modelling approach, we estimated the hazard ratio of SARS-CoV-2 infection according to age and number of all household children (age < 18 years) relative to only the children aged 6 years, to explore relative effects of child age. In this analysis, each adult contributed with a number of observations equal to their number of children. The model included a restricted cubic spline term with four knots (located at the 5th, 35th, 65th and 95th percentile of the age distribution) for child age, a three-level variable for number of children (one, two, or three or more children) and a robust variance structure and was adjusted for the covariates included in the previous analyses. The Bayesian information criterion was used to choose between a model with an interaction term for child age and number of household children and a model with additive effect.

## Results

In our cohort of 3,078,507 adults living in Denmark aged 18 to 60 years, 450,007 (14.6%) lived in households with young children aged 10 months to 5 years, while 2,628,500 (85.4%) lived in households without young children (Table 1). Adults living with young children were, on average, younger (median age: 35 vs 42 years) and more often female (54% vs 49%) than adults not living with young children. For both groups, the most common household type consisted of two adults (86% among adults living with young children and 49% among adults not living with young children). For the group of adults in households with young children, 76%, 23% and 1% lived with one, two, or three or more young children, respectively. For adults in households with any children under 18 years, 35%, 48% and 18% lived with one, two, or three or more children, respectively. Medical comorbidities, as defined from nationwide hospital diagnostic codes, were more common among individuals not living in households with young children, except for inflammatory bowel disease, which was slightly more common among individuals living with younger children.

**Table 1:** Baseline characteristics of the cohort of adults by household type, Denmark, 27 February 2020–26 February 2021 (n = 3,078,507)

Characteristic	Adults living in households with young children (n = 450,007)		Adults living in households without young children (n = 2,628,500)	
	n	%	n	%
Median age in years (25th to 75th percentile)	35 (31–39)		42 (27–51)	
Female sex	242,328	53.8	1,287,512	49.0
Household number of children aged 10 months to 5 years				
0	NA		2,628,500	100.0
1	341,198	75.8	NA	
2	104,150	23.1		
≥ 3	4,659	1.0		
Household number of children in total (< 18 years)				
0	NA		1,964,096	74.7
1	157,264	34.9	323,293	12.3
2	214,085	47.6	272,237	10.4
≥ 3	78,658	17.5	68,874	2.6
Household number of adults				
1	30,867	6.9	616,533	23.5
2	387,163	86.0	1,295,057	49.3
≥ 3	31,977	7.1	716,910	27.3
Ethnicity^a^				
Danish	351,732	78.2	2,186,550	83.2
Western	31,996	7.1	172,667	6.6
Non-Western	66,092	14.7	268,411	10.2
Missing information	187	0.0	872	0.0
Comorbidities				
Asthma	11,640	2.6	71,534	2.7
Chronic pulmonary disease (incl. COPD)	1,256	0.3	25,158	1.0
Cardiovascular disease	2,224	0.5	42,888	1.6
Diabetes mellitus	4,0194	0.9	51,708	2.0
Inflammatory bowel disease	6,045	1.3	31,629	1.2
Malignancy	5,095	1.1	63,744	2.4
Renal failure	2023	0.4	18,340	0.7

COPD: chronic obstructive pulmonary disease; NA: not applicable.

^a^ Ethnicity was defined according to the definition used by Statistics Denmark [15]. Missing information was imputed to Danish ethnicity for adjustment.

When investigating risk of SARS-CoV-2 infection in adults living in households with young children compared with adults living in households without young children, we found overall an adjusted hazard ratio of 1.10 (95% CI: 1.08–1.12) for SARS-CoV-2 infection (Table 2). When stratifying by number of young children in the household we found an adjusted hazard ratio for SARS-CoV-2 infection of 1.08 (95% CI: 1.06–1.10), 1.16 (95% CI: 1.12–1.20) and 1.38 (95% CI: 1.18–1.61) for living in a household with one, two, or three or more children, respectively, compared with individuals in households with no young children (p < 0.0001 for trend).

**Table 2:** Hazard ratio of SARS-CoV-2 infection in adults by household type and number of young children, Denmark, 27 February 2020–26 February 2021 (n = 3,078,507)

Household type	SARS-CoV-2-positive adults	Adults in total	Hazard ratio of SARS-CoV-2 infection (95% CI)	
			Crude^a^	Adjusted^b^
Household without young children	110,069	2,628,500	1 (reference)	1 (reference)
Household with young children (any)	19,555	450,007	1.08 (1.06–1.10)	1.10 (1.08–1.12)
1	14,735	341,198	1.07 (1.05–1.10)	1.08 (1.06–1.10)
2	4,570	104,150	1.10 (1.06–1.14)	1.16 (1.12–1.20)
≥ 3	250	4,659	1.35 (1.16–1.57)	1.38 (1.18–1.61)

CI: confidence interval; SARS-CoV-2: Severe acute respiratory syndrome coronavirus 2.

^a^ Crude: age and sex adjusted only.

^b^ Adjusted: further adjusted for urbanicity, ethnicity and comorbidities. P for trend < 0.0001.

Investigating the risk of SARS-CoV-2 hospitalisation among SARS-CoV-2-positive adults by household status, we found a non-significant decreased risk of SARS-CoV-2 hospitalisation among adults living in households with any number of young children compared with adults living without young children in the household (adjusted hazard ratio = 0.97; 95% CI: 0.88–1.08) (Table 3). When stratifying by number of young children in the household, we found an adjusted hazard ratio of 1.02 (95% CI: 0.91–1.13) and 0.83 (95% CI: 0.69–1.00) of SARS-CoV-2 hospitalisation for adults living with one, or two or more children, respectively, compared with adults in households with no young children (p = 0.50 for trend).

**Table 3:** Hazard ratio of SARS-CoV-2 hospitalisation in SARS-CoV-2-positive adults by household type and number of young children, Denmark, 27 February 2020–26 February 2021 (n = 129,363)

Household type	Adults hospitalised	SARS-CoV-2-positive adults in total	Hazard ratio of SARS-CoV-2 hospitalisation (95% CI)	
			Crude^a^	Adjusted^b^
Household without young children	4,003	109,827	1 (reference)	1 (reference)
Household with young children (any)	620	19,536	1.00 (0.91–1.11)	0.97 (0.88–1.08)
1	494	14,722	1.05 (0.94–1.17)	1.02 (0.91–1.13)
≥ 2	126	4,814	0.84 (0.70–1.02)	0.83 (0.69–1.00)

CI: confidence interval; SARS-CoV-2: Severe acute respiratory syndrome coronavirus 2.

^a^ Crude: age and sex adjusted only.

^b^ Adjusted: further adjusted for urbanicity, ethnicity and comorbidities. P for trend = 0.50.

We performed a series of sensitivity analyses for which detailed information is provided in Supplementary Tables S2–S8. In the sensitivity analyses, we considered the role of adult age, sex and time period of testing (Supplementary Table S2). We found the relative SARS-CoV-2 infection risk to be highest among adults aged 30–39 years sharing a household with young children (adjusted hazard ratio = 1.17; 95% CI: 1.14–1.20) and lowest among adults aged 40–59 years sharing a household with young children (adjusted hazard ratio = 1.00; 95% CI: 0.96–1.04). In addition, we found evidence of a significant, but small, difference by sex, with higher risk of SARS-CoV-2 infection among men compared with women (p < 0.0001 for interaction). We evaluated different definitions of young children and household children, and presence of older and younger children in the same household (Supplementary Tables S3-S6). While we found no major role of slight changes in the age span definition or the definition of household children, we found strong evidence of increased SARS-CoV-2 infection risk among adults with older children in the household, regardless of whether young children were present in the household (adjusted hazard ratio = 1.34; 95% CI: 1.31–1.38) or not (adjusted hazard ratio = 1.32; 95% CI: 1.29–1.34). In households with only young children, a pattern of increased risk of infection by increasing number of young children persisted. In addition, we examined the role of the number of adults in the household (Supplementary Table S7). When stratifying by number of household adults, we found a heterogeneous pattern, whereby the smallest increase in hazard ratio of SARS-CoV-2 infection was in households with two adults (adjusted hazard ratio = 1.05; 95% CI: 1.03–1.08), while we observed the largest increased risk in households with one adult (adjusted hazard ratio = 1.26; 95% CI: 1.19–1.33). Finally, as a last sensitivity analysis, we investigated SARS-CoV-2 testing intensity by household type (Supplementary Table S8). We found that adults in households with young children had a 7% increased testing rate compared with adults in households without young children. However, adjusting for testing intensity had minimal impact on our main finding, with an adjusted hazard ratio of SARS-CoV-2 infection of 1.08 (95% CI: 1.06–1.11) for adults in households with any number of young children compared with adults in a household without young children, when adjusted for tests within the latest 60 days. It should be mentioned that during the study period, testing of children in nurseries, pre-schools and schools was not a requirement for attendance. However, from March 2021 (after the study period), regular self-testing was encouraged for school attendance.

To illustrate relative effects of child age, we plotted cubic splines of SARS-CoV-2 infection risk in adults living in households with children by child age and total number of children in the household relative to having one child aged 6 years (Figure). Overall, older child age and larger number of children was associated with an additive increased hazard ratio of infection.

**Figure:**
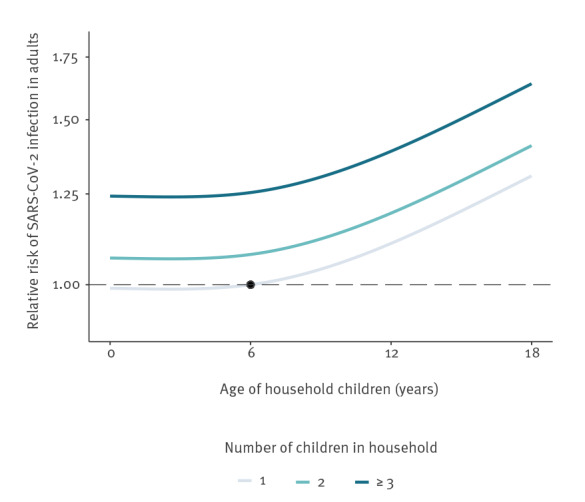
Hazard ratio of SARS-CoV-2 infection in adults living in households with children, by child age and total number of children in the household relative to having one child aged 6 years, Denmark, 27 February 2020–26 February 2021 (n = 3,078,507)

SARS-CoV-2: Severe acute respiratory syndrome coronavirus 2.

Adjusted for age, sex, urbanicity, ethnicity, and comorbidities.

## Discussion

In a nationwide cohort study of all adults in Denmark aged 18 to 60 years, living in households with young children was associated with a small, but significant, increased risk of SARS-CoV-2 infection, compared with adults living in households with no young children, adjusting for potential confounders. The risk of infection was amplified with increasing number of young children living in the household and with older children living in the household. Overall, the results suggest an increased SARS-CoV-2 infection risk in adults living with young children, but lower compared to living with older children.

Our study is, to our knowledge, the first study of household characteristics and SARS-CoV-2 infection risk enrolling an entire population of more than 3 million individuals. Furthermore, the study probably captured the vast majority of SARS-CoV-2 infections in Denmark during most of the study period, as the healthcare system in Denmark provided free and easily assessable testing for all Danish inhabitants, regardless of COVID-19 symptoms, during the study period. This is reflected in the low positivity rate of SARS-CoV-2 tests of 2% from late April 2020 to the end of follow-up in February 2021 (Supplementary Figure S1).

Our study has a number of limitations. We did not include information on SARS-CoV-2 testing of young children. However, young children are often asymptomatic or present with mild symptoms of infection [16], thus inclusion of this information would introduce an undesirable bias of health-seeking behaviour into our analyses. In addition, we did not assess clinical symptoms of index cases. Nevertheless, we do not suspect differential symptoms between adults living with and without young children and therefore do not suspect that this aspect of a SARS-CoV-2 infection would confound the propensity for testing and detection of SARS-CoV-2. Furthermore, we did not have serological measurements of pre-existing immunity to HCoV, which could offer direct biological evidence of pre-existing immunity against SARS-CoV-2 following prior exposure to HCoV, but co-habitation with young children has previously been considered a reasonable proxy for recent exposure to HCoV [6]. Lastly, in the study design, we defined the cohort member’s exposure status (i.e. living with or without young children) according to 1 January 2020, which could lead to some exposure misclassification. Nevertheless, our sensitivity analysis of child age definitions had roughly similar results, indicating that the findings are robust to minor changes in inclusion criteria of the exposure.

Our findings show that having young children in one’s household was associated with a slightly increased risk of SARS-CoV-2 infection. The association could be a result of social contacts among adults living in households with young children (e.g. more adults in the household, contact to day care facilities, or close contact to parents of playmates) or of infection brought into the household by the young children. Nevertheless, when we stratified by number of adults in the household we found no indication that an increased number of adults in the household was the driving force behind increased risk of SARS-CoV-2 infection. However, we found a heterogeneous pattern according to which the lowest hazard ratio of infection was in households with two adults, indicating that social circumstances (e.g. having children who live interchangeably in another household), and not the household number of adults per se, is the most important factor in determining household infection risk. Furthermore, our analyses also show an increasing infection risk with increasing number of young children. Still, compared with older children, the increased infection risk from living with young children within the household is relatively small. It is therefore important to weigh this relatively small increased risk of SARS-CoV-2 transmission against the many benefits of young children attending day care facilities and having playdates.

The lower hazard ratio of SARS-CoV-2 infection when living with younger children compared with living with older children is intriguing given that parents are likely to have more close contact with younger children, especially if they become ill. Nevertheless, our finding is in line with conclusions from contact tracing and population screening studies, which show lower susceptibility, and potentially limited transmissibility, of SARS-CoV-2 infection among younger children compared with older children [4]. One possible explanation for the association is that the peak viral load of SARS-CoV-2 increases with age [17]. Another potentially contributing factor is that illness duration is longer in older children than in younger children [18]. However, our findings indicate that previous exposure to HCoV does not explain the difference in effects between younger and older children, since living with multiple younger children, which would be associated with more frequent exposure to seasonal HCoV, in itself is associated with increased SARS-CoV-2 infection risk. Taken together, our study therefore suggests an increased risk of SARS-CoV-2 infection from contact with older compared with younger children that is not explained by exposure to seasonally spreading HCoV.

Our findings are generally in line with a similar study from the OpenSAFELY cohort, composed of 12 million adults in England with information on number of children in households gathered from primary care records [19]. As opposed to previous studies, we were also able to investigate the severity of SARS-CoV-2 infection in a complete population, using information on hospital admissions of all SARS-CoV-2 positive cases. We did not find any indication of a difference in the hazard ratio of hospitalisation among individuals living with young children compared with individuals living without. Nevertheless, this analysis had limited power because few adults with young children were hospitalised in Denmark. Furthermore, the OpenSAFELY study, which is based on a large population sample, found an increased relative risk of COVID-19 hospital admission among individuals living with younger children (defined as 0–4 years in their sensitivity analysis) during the second wave of the pandemic. Taken together with the previous observation that parents are generally healthier than non-parents [20], beyond what we in our study can capture by registered clinical comorbidities, and that parents might be more prone to avoid hospitalisation, we cannot exclude the possibility that living with younger children can be associated with a small increased risk of severe SARS-CoV-2 infection, which could qualify for hospitalisation.

## Conclusion

We found no evidence of a reduced risk of SARS-CoV-2 infection in adults living with young children. On the contrary, we found a significant, slightly increased hazard ratio of SARS-CoV-2 infection. Our study suggests that living with young children, and thereby being frequently exposed to HCoV, does not offer substantial protection against SARS-CoV-2 infection, but on the contrary slightly increases an adult's risk of SARS-CoV-2 infection.

## Supplementary Data

705000 Supplement
