# Supplementary material for: SARS-CoV-2 infection in households with and without young children: Nationwide cohort study, Denmark, 27 February 2020 to 26 February 2021
Source: Euro Surveill. 2022 Aug 11;27(32):2101096. doi: 10.2807/1560-7917.ES.2022.27.32.2101096 (PMC9373601; doi:10.2807/1560-7917.ES.2022.27.32.2101096)
Supplement: Supplement [file 21-01096_HUSBY_supplement.pdf]

Supplementary material to "**SARS-CoV-2 infection in households with and without young children: Nationwide cohort study in Denmark from February 27, 2020 to February 26, 2021**"

This supplementary material is hosted by Eurosurveillance as supporting information alongside the article *SARS-CoV-2 infection in households with and without young children: Nationwide cohort study in Denmark from February 27, 2020 to February 26, 2021*, on behalf of the authors, who remain responsible for the accuracy and appropriateness of the content. The same standards for ethics, copyright, attributions and permissions as for the article apply. Supplements are not edited by Eurosurveillance and the journal is not responsible for the maintenance of any links or email addresses provided therein.

Table of contents:

Figure S1. SARS-CoV-2 epidemic in Denmark. 2

Table S1. Diagnostic codes used for ascertainment of comorbidities.......... 3

Table S2. Relative risk of SARS-CoV-2 infection in adults by household type and number of young children, by adult age, gender, and time period. 4

Table S3. Relative risk of SARS-CoV-2 infection in adults by household type and number of young children, by age span definition. 5

Table S4. Relative risk of SARS-CoV-2 infection in adults by household type and number of young children, by exposure with all young children in household and out-of-household. 6

Table S5. Relative risk of SARS-CoV-2 infection in adults by household type and number of young and older children in household. 7

Table S6. Relative risk of SARS-CoV-2 infection in adults living in household with young children, and not older children, by number of young children.......... 8

Table S7. Relative risk of SARS-CoV-2 infection in adults by household type and by number of adults in household.......... 9

Table S8. Relative risk (incidence rate ratio) of test for SARS-CoV-2 in adults by household type. 10

Supplementary Figures

Figure S1. SARS-CoV-2 epidemic in Denmark. Daily SARS-CoV-2 test positivity rate (A), cases (B), and hospital admissions (C) up to February 26, 2021.

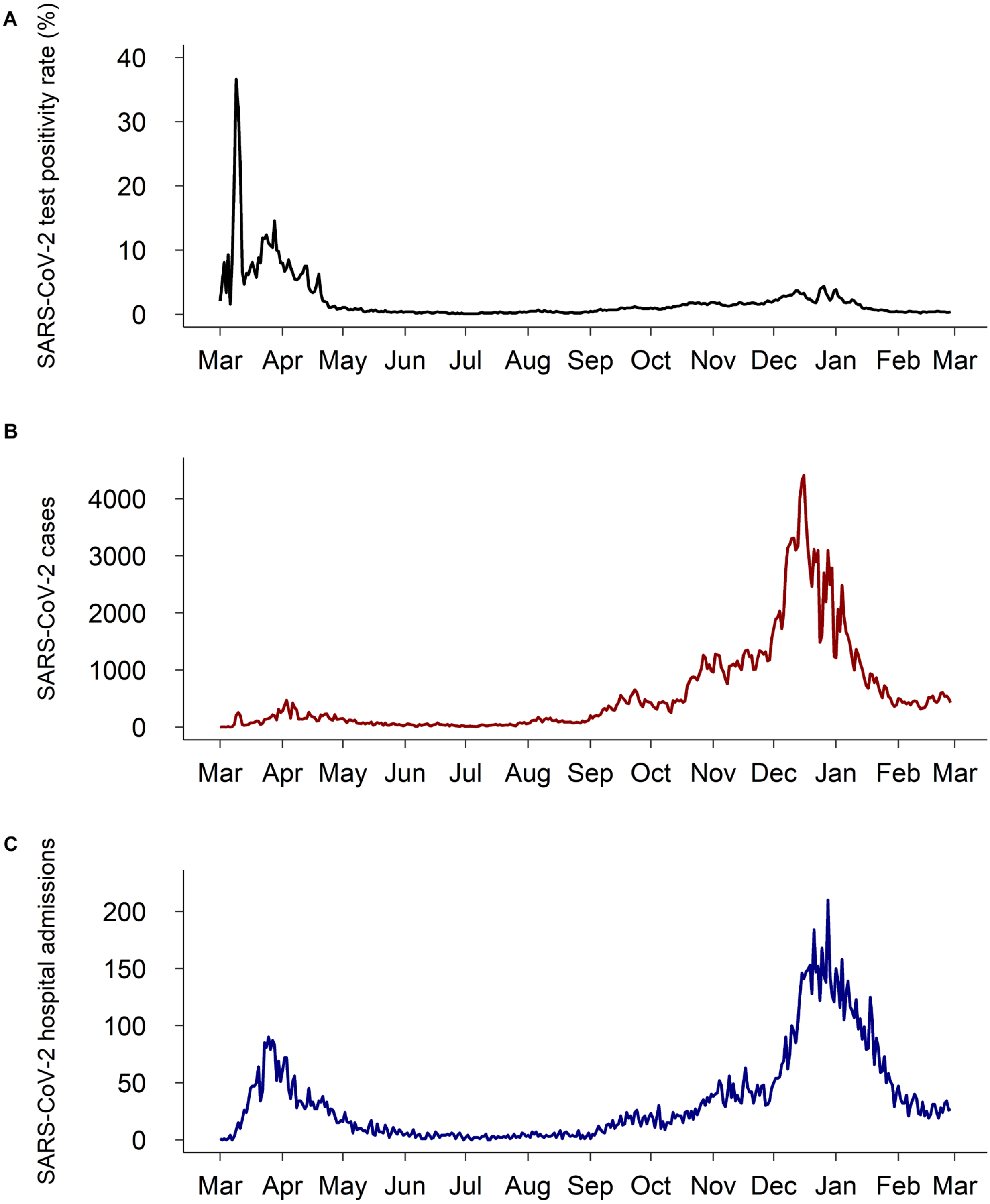

Supplementary Tables

**Table S1.** Diagnostic codes used for ascertainment of comorbidities.	
Comorbidity	**ICD-10 codes**
Asthma	J45-J46
Chronic pulmonary disease (incl. COPD)	J40-J44, J47, J60–J67, J68.4, J70.1, J70.3, J84.1, J92.0, J96.1, J98.2, J98.3
Cardiovascular disease	I11.0, I13.0, I13.2, I21-I23, I48, I50x,
Diabetes mellitus	E10.0, E10.1, E10.9, E11.0, E11.1, E11.9
Inflammatory bowel disease	K50x, K51x
Malignancy	C00–C75
Renal failure	I12, I13, N00–N05, N07, N11, N14, N17–N19, Q61

**Table S2.** Relative risk of SARS-CoV-2 infection in adults by household type and number of young children, by adult age, gender, and time period.				
	**Adult cases with young children / Adults in total**	**Adult cases without young children / Adults in total**	**Relative risk of SARS-CoV-2 infection** *hazard ratio (95% CI)*	
			**Crude[a]**	**Adjusted[b]**
**Age (years)[c]**				
18-29	3343 / 75,053	40,642 / 810,298	0.96 (0.92,1.00)	1.08 (1.03,1.12)
30-39	11,988 / 278,167	14,682 / 385,721	1.12 (1.09,1.15)	1.17 (1.14,1.20)
40-59	4,224 / 96,787	54,745 / 1,432,481	1.13 (1.09,1.17)	1.00 (0.96,1.04)
**Gender[d]**				
Female	10,757 / 242,328	57,142 / 1,287,512	1.04 (1.02,1.06)	1.07 (1.04,1.09)
Male	8,798 / 207,679	52,927 / 1,340,988	1.14 (1.11,1.17)	1.14 (1.12,1.17)
**Time period[e]**				
February 27-March 26 (pre-lockdown)	166 / 445,007	1,101 / 2,628,500	0.92 (0.78,1.10)	0.94 (0.79,1.11)
March 27-April 28 (lockdown)	684 / 449,624	3,876 / 2,624,807	1.08 (0.98,1.18)	1.10 (1.00,1.20)
April 29-June 30 (early reopening)	366 / 448,829	1,802 / 2,619,375	1.24 (1.09,1.41)	1.26 (1.11,1.44)
July 1-November 30 (late reopening)	6,251 / 448,067	37,240 / 2,612,854	1.02 (0.99,1.06)	1.04 (1.00,1.07)
December 1, 2020 -February 27, 2021	12,088 / 440,639	66,050 / 2,563,132	1.11 (1.09,1.14)	1.13 (1.10,1.16)

[a]Crude: Age and gender adjusted only.

[b]Adjusted: Furthermore adjusted for urbanicity, ethnicity, and comorbidities.

[c] P-value for interaction <0.0001 in adjusted analysis.

[d] P-value for interaction <0.0001 in adjusted analysis.

[e] P-value for interaction = 0.05 in adjusted analysis. During the study period nurseries and pre-schools (ages 0-5 years) were closed from March 12, 2020 to April 15, 2020. Early school years (primarily ages 6-10 years) were closed from March 12, 2020 to April 15, 2020, and again from December 21, 2020 to February 8, 2021. Middle school years (primarily ages 11-14 years) were closed from March 12, 2020 to May 18, 2020, and again from December 9, 2020 to April 6, 2021. Late school years (ages 15-16 years) were closed from March 12, 2020 to May 18, 2020 and again from December 9, 2020 to April 2021.

**Table S3.** Relative risk of SARS-CoV-2 infection in adults by household type and number of young children, by age span definition.				
**Age span definition**	**Adult cases with young children / Adults in total**	**Adult cases without young children / Adults in total**	**Relative risk of SARS-CoV-2 infection** *hazard ratio (95% CI)*	
			**Crude**[a]	**Adjusted**[b]
1-3 years	13,362 / 309,739	116,262 / 2,768,768	1.05 (1.03,1.08)	1.08 (1.05,1.10)
10 months – 5 years	19,555 / 450,007	110,069 / 2,628,500	1.08 (1.06,1.10)	1.10 (1.08,1.12)
0-9 years	28,565 / 658,742	101,059 / 2,419,765	1.10 (1.08,1.12)	1.12 (1.10,1.14)

[a]Crude: Age and gender adjusted only.

[b]Adjusted: Furthermore adjusted for urbanicity, ethnicity, and comorbidities.

**Table S4.** Relative risk of SARS-CoV-2 infection in adults by household type and number of young children, by exposure with all young children in household and out-of-household.				
**Household children definition**	**Adult cases with young children / Adults in total**	**Adult cases without young children / Adults in total**	**Relative risk of SARS-CoV-2 infection** *hazard ratio (95% CI)*	
			**Crude**[a]	**Adjusted**[b]
All legally parented young children	20,712 / 480,808	108,912 / 2,597,699	1.07 (1.05,1.09)	1.09 (1.07,1.11)
All household co-living young children	20,927 / 475,892	108,697 / 2,602,615	1.10 (1.07,1.12)	1.09 (1.07,1.12)
Only legally parented and co-living household young children	19,555 / 450,007	110,069 / 2,628,500	1.08 (1.06,1.10)	1.10 (1.08,1.12)

[a]Crude: Age and gender adjusted only.

[b]Adjusted: Furthermore adjusted for urbanicity, ethnicity, and comorbidities.

**Table S5.** Relative risk of SARS-CoV-2 infection in adults by household type and number of young and older children in household.			
**Household type**	**Adult cases / Adult in total**	**Relative risk of SARS-CoV-2 infection** *hazard ratio (95% CI)*	
		**Crude**[a]	**Adjusted**[b]
Household without any children	80,119 / 1,964,096	1 (ref.)	1 (ref.)
Household with young children and without older children (≥ 6 years)	9,824 / 242,268	1.07 (1.04,1.10)	1.11 (1.08,1.14)
Household with young children and with older children (≥ 6 years)	9,725 / 207,739	1.31 (1.28,1.35)	1.34 (1.31,1.38)
Household without young children and with older children (≥ 6 years)	29,948 / 664,404	1.27 (1.25,1.30)	1.32 (1.29,1.34)

[a]Crude: Age and gender adjusted only.

[b]Adjusted: Furthermore adjusted for urbanicity, ethnicity, and comorbidities.

Table S6. Relative risk of SARS-CoV-2 infection in adults living in household with young children, and not older children, by number of young children.

Number of young children	Adult cases / Adults in total	Relative risk of SARS-CoV-2 infection *hazard ratio (95% CI)*	
		**Crude**[a]	**Adjusted**[b]
1	6,183 / 157,264	1 (ref.)	1 (ref.)
2	3,437 / 81,079	1.10 (1.04,1.16)	1.19 (1.13,1.25)
3+	208 / 3,925	1.38 (1.17,1.64)	1.46 (1.23,1.73)

[a]Crude: Age and gender adjusted only.

[b]Adjusted: Furthermore adjusted for urbanicity, ethnicity, and comorbidities.

**Table S7.** Relative risk of SARS-CoV-2 infection in adults by household type and by number of adults in household.				
**Number of adults in household**	**Adult cases with young children / Adults in total**	**Adult cases without young children / Adults in total**	**Relative risk of SARS-CoV-2 infection** *hazard ratio (95% CI)*	
			**Crude**[a]	**Adjusted**[b]
1	1,352 / 30,867	19,884 / 616,533	1.27 (1.20,1.34)	1.26 (1.19,1.33)
2	16,186 / 387,136	52,527 / 1,295,057	1.03 (1.00,1.05)	1.05 (1.03,1.08)
3+	2,017 / 31,977	37,658 / 716,910	1.24 (1.17,1.32)	1.12 (1.06,1.19)

[a]Crude: Age and gender adjusted only.

[b]Adjusted: Furthermore adjusted for urbanicity, ethnicity, and comorbidities.

**Table S8.** Relative risk (incidence rate ratio) of test for SARS-CoV-2 in adults by household type.						
**Household type**	**Adults in total**	**Tested adults**	**Number of tests**	**Incidence test rate (1000 persons/week)**	**Relative risk of test for SARS-CoV-2** *IRR (95% CI)*	
					**Crude**[a]	**Adjusted**[b]
Household without young children	2,628,500	2,051,353	8,859,965	64.6	1 (ref.)	1 (ref.)
Household with young children (any)	450,007	378,837	1,597,991	67.9	1.06 (1.06,1.07)	1.07 (1.07,1.07)
1	341,198	286,951	1,205,236	67.6	1.06 (1.05,1.06)	1.06 (1.06,1.06)
2	104,150	88,179	377,742	69.4	1.10 (1.09,1.10)	1.10 (1.10,1.11)
3+	4,659	3,707	15,013	61.6	0.97 (0.96,0.99)	1.01 (0.99,1.03)

[a]Crude: Age and gender adjusted only.

[b]Adjusted: Furthermore adjusted for urbanicity, ethnicity, and comorbidities.
